# Parity and Mortality: An Examination of Different Explanatory Mechanisms Using Data on Biological and Adoptive Parents

**DOI:** 10.1007/s10680-018-9469-1

**Published:** 2018-02-21

**Authors:** Kieron Barclay, Martin Kolk

**Affiliations:** 1 Max Planck Institute for Demographic Research, 18057 Rostock, Germany; 2 Department of Social Policy, London School of Economics and Political Science, London, WC2A 2AE UK; 3 Department of Sociology, Stockholm University, 106 91 Stockholm, Sweden; 4 Centre for the Study of Cultural Evolution, Stockholm University, 106 91 Stockholm, Sweden; 5 Institute for Futures Studies, Holländargatan 13, 101 31 Stockholm, Sweden

**Keywords:** Parity, Mortality, Adoption, Register data, Sweden

## Abstract

A growing literature has demonstrated a relationship between parity and mortality, but the explanation for that relationship remains unclear. This study aims to pick apart physiological and social explanations for the parity–mortality relationship by examining the mortality of parents who adopt children, but who have no biological children, in comparison with the mortality of parents with biological children. Using Swedish register data, we study post-reproductive mortality amongst women and men from cohorts born between 1915 and 1960, over ages 45–97. Our results show the relative risks of mortality for adoptive parents are always lower than those of parents with biological children. Mortality amongst adoptive parents is lower for those who adopt more than one child, while for parents with biological children we observe a U-shaped relationship, where parity-two parents have the lowest mortality. Our discussion considers the relative importance of physiological and social depletion effects, and selection processes.

**Electronic supplementary material:**

The online version of this article (10.1007/s10680-018-9469-1) contains supplementary material, which is available to authorized users.

## Introduction

This study contributes to the literature concerning the relationship between parity and mortality by examining the mortality of women and men who adopt children in contemporary Sweden. By examining the mortality of mothers and fathers who adopt children but who have no biological children of their own we hope to partially adjudicate between the various physiological and social theories that have been proposed for the relationship between parity and mortality. More specifically, we argue that since theories based on the physiological drain of childbearing concerning the hypothesised parity–mortality relationship do not apply to adoptive parents, we will be able to look at the relative contribution of the posited social mechanisms relating parity to post-reproductive mortality. In this study we use the term parity uniformly for both women and men, and for both adoptive parents and parents with biological children.

Recent meta-analyses of studies using contemporary data on the relationship between biological parity and all-cause mortality show that there is a J-shaped relationship between the two variables (Högnäs et al. 2017; Zeng et al. 2016); mortality is elevated for childless men and women, is lowest for parity-two mothers and fathers, and increases relative to parity-two parents at higher parities (Kvåle et al. 1994; Doblhammer 2000; Manor et al. 2000; Hurt et al. 2004; Grundy and Tomassini 2005; Koski-Rahikalla et al. 2006; Grundy 2009; Jaffe et al. 2009, Dior et al. 2013). However, some studies, though not all (Koski-Rahikalla et al. 2006), using data from the Nordic region show that parity-three plus women do not have higher mortality relative to parity-two women (Hinkula et al. 2006; Grundy and Kravdal 2008, 2010). Several studies that have taken care to adjust their analyses for socioeconomic status have shown that the relationship between parity and mortality differs between socioeconomic groups (Dribe 2004; Hurt et al. 2006; Grundy and Kravdal 2010). Fewer studies have addressed the relationship between parity and mortality for men than for women. In contemporary populations in high-income societies the relationship between parity and mortality is generally similar for both sexes (Grundy and Kravdal 2008, 2010; Barclay et al. 2016).

There are several different explanatory models concerning the relationship between parity and mortality. We will here discuss six different explanations, which are biomedical models, evolutionary models, maternal depletion models, social support models, selection models (Alter et al. 2007), and lifestyle changes induced by entry into parenthood. The first two groups of explanations, biomedical, and evolutionary models, may be categorised as physiological explanations for the hypothesised parity–mortality relationship, whereas the social support, selection, and lifestyle change explanations can be classified as social explanations. Maternal depletion models describe depletion by both physiological and social mechanisms and therefore defy this binary classification. It should be noted that many of the explanations overlap and that a single physiological or social phenomenon causing a relationship between mortality and parity often could be classified as belonging to several different theoretical explanations.

Biomedical models address the physiological processes that are triggered by pregnancy, childbirth, and lactation, which have been linked to increased risks of suffering from certain health problems and a diminished risk of suffering from others, such as cancers of the breast, ovary, and uterus (Ellison 2001; Grundy and Kravdal 2010). Key amongst these processes is the role that ovarian hormones play. Ovarian hormones, particularly progesterone and oestrogen, stimulate cell growth, including the growth of cancerous tissues (Kelsey et al. 1993). Women are amenorrhoeic during pregnancy and lactation. As a result, women with children and who breastfeed experience fewer menstrual cycles than childless women, and repeated childbearing particularly reduces the cumulative exposure to progesterone and oestrogen. Studies on cause-specific mortality indicate that higher parity women have a lower risk of breast, uterine, and ovarian cancer (Merrill et al. 2005; Barclay et al. 2016), which is consistent with the hypothesised mechanisms.

A dominant theory within the group of evolutionary models is the disposable soma theory (Kirkwood and Holliday 1979; Ellison 2001). The disposable soma theory posits a direct trade-off between childbearing and longevity for women, where having more children should decrease longevity. The maternal depletion model bears similarities to disposable soma theory in emphasising that childbearing is costly to the mother in terms of the direct physiological drain of childbearing. However, the maternal depletion model also emphasises the emotional and social stress that childrearing has the potential to incur. While termed the maternal depletion model, this social depletion mechanism certainly also has the potential to apply to fathers, in particular in a context in which fathers are involved in a significant way in childrearing such as contemporary Sweden. This social depletion may also include the indirect costs of childbearing in lost earnings and the potential impact that may have on health. However, the labour market consequences of parenthood are likely to vary by gender, with mothers typically penalised in the labour market (Correll et al. 2007; Aisenbrey et al. 2009) and men benefitting from the fatherhood premium (Bygren and Gähler 2012; Killewald 2013). Nevertheless, when earnings are shared at the household level the consequences of earnings loss amongst women are likely to be heavily tempered.

The three groups of models that more exclusively emphasise social mechanisms, though those are also somewhat touched upon by maternal depletion theory, are the social support models, selection models, and behavioural explanations. The social support model emphasises the potential support, both social and financial, that children can provide to parents in their post-reproductive years. Recent research has shown that the socioeconomic status of children is associated with parental mortality (Torssander 2013; Friedman and Mare 2014; Zimmer et al. 2016), which may be attributable to the extent to which children are able to direct time and resources to help their ageing parents. Net of the socioeconomic status of the children, a greater number of children might also be associated with greater social support for the parents as they age, as this increases the likelihood that some of them may live nearby and be willing to set aside the time to help the parents. Research also consistently shows that patterns of caregiving are gendered, and daughters are more likely than sons to live nearby, as well as to care for, ailing parents (Rossi and Rossi 1990; Fors and Lennartsson 2008).

The selection model addresses the fact that both limited childbearing and longevity may be confounded by factors such as education, class, and income, as these socioeconomic factors are also associated with mortality (Torssander and Erikson 2010). Considering selection processes related to socioeconomic status and health separately facilitates a better understanding of the relationship between parity and mortality. Socioeconomic selection might exist if groups with different socioeconomic statuses have different desires and outcomes in terms of number of children. For example, educational level is associated with childbearing behaviour and is also associated with mortality, and families with a large number of children are likely to be negatively selected on certain socioeconomic characteristics (Andersson et al. 2009). Childless individuals are also on average more common in highly disadvantaged groups. Adoption propensities are also likely to differ by socioeconomic background. This is particularly the case as international adoptions are associated with substantial economic costs, which the parents themselves have to bear to a large extent. Health selection is also likely to play an important role in explaining the relationship between parity and mortality. First, healthy individuals are more able to attract a partner (Lillard and Panis 1996), an important precondition for having children. Amongst those who have children, a large number of children might also be evidence of good health, and contrastingly, childlessness and low fecundity evidence of poor health. Health selection is particularly important with respect to adoption. In Sweden, as in many other countries, individuals seeking to adopt need to go through a rigorous process to assess their perceived suitability as parents by the adoption authorities. This assessment process includes structured interviews, at least one home visit, character references, various background checks, and disclosures of medical history (Socialstyrelsen 2009). The Swedish adoption authorities screen prospective parents on wide range of characteristics that fall under three categories: (1) family and environmental factors, (2) parenting capacity, and (3) a child’s developmental needs (Socialstyrelsen 2009, pages 50–51).

Of particular relevance to this study are the requirements concerning the physical and mental health of potential adoptive parents. The Swedish adoption agency requires that adoptive parents should be physically and mentally capable of performing all the functions expected of a parent throughout the childhood and teenage years of the child they adopt (Socialstyrelsen 2009). Adoptive parents must supply the adoption agency with a health statement and a medical certificate from a doctor, a disclosure of the past 10 years of social insurance receipts that might reveal periods of sickness absence from work, and medical details about mental health if there are any concerns about that dimension. If the social worker responsible for the evaluation assesses that the applicant’s health history would influence their health on a day-to-day basis over the next two decades or so, that applicant for adoption is likely to be refused. Social workers also assess observable physical health and lifestyle. Applicants who are obese or underweight are likely to fail the assessment. Patterns of alcohol consumption and smoking are also important factors in the evaluation. Other factors such as being in a stable and supportive relationship, having sufficient financial resources and a stable job, having a supportive social network more generally, being integrated into the community, and other personal qualities are also assessed. Broadly speaking, these factors are amongst the most important social determinants of health (Link and Phelan 1995; Smith and Christakis 2008). Nevertheless, we should consider that adoptive parents may have chosen to adopt because of infertility, which may indicate a lower level of underlying health. However, given the careful assessment of medical history and observable physical and mental health, adoptive parents in Sweden are atypically healthy, robust, and stable individuals, comparable to other healthy vanguard groups (Mehta and Myrskylä 2017).

Although the contemporary adoption process in Sweden is long and arduous, the process was substantially less selective earlier in the twentieth century. Prior to the 1970s, domestic adoptions were far more frequent than transnational adoptions, and it was also relatively common to adopt a child from relatives if some unfortunate event had befallen the biological parents of the child. Since domestic adoptions became very uncommon after the adoption process became highly selective, this provides an opportunity to distinguish between adoptive parents with no biological children who went through a rigorous selection on a healthy and well-rounded lifestyle, and adoptive parents who were not required to go through that procedure. By comparing the mortality of adoptive parents who adopted children domestically versus adoptive parents who adopted children transnationally, we will be able to assess the degree to which positive selection by the adoption authorities influences the patterns of mortality for adoptive parents. Since those who adopted transnationally had to undergo a much more selective screening process than those who adopted domestically, we anticipate the mortality of adoptive parents who adopted domestically will be similar to the mortality of biological parents if physiological mechanisms do not play an important role in explaining the relationship between parity and mortality. Furthermore, by carefully examining the mortality of adoptive parents who did undergo the screening process, we will be able to examine how number of children is related to mortality net of health factors that are otherwise difficult to measure, as all those who adopt transnationally are required to pass a certain health threshold.

In addition to selection into parenthood based on health characteristics, it is also important to consider how the presence of children may influence lifestyle. In general, studies suggest that entry into parenthood increases the likelihood that individuals behave in a more responsible manner, and this is particularly true for men. The new responsibilities that parenthood brings may discourage heavy alcohol consumption (Chilcoat and Breslau 1996) and help smokers find the resolve to quit the habit (McDermott et al. 2006). More generally, the obligations of childrearing increase domesticity, and there is also evidence that parenthood encourages greater integration into the local community (Knoester and Eggebeen 2006). The domesticating nature of parenthood is likely to have particularly protective health benefits for men, who are generally more likely to engage in risky health behaviours than women, and particularly so when they are without children or a partner (Nock 1998). Since childrearing is often accompanied by a stable relationship, the protective health benefits that partnership, cohabitation, and marriage bring will also overlap with the behavioural changes that accompany parenthood (Umberson 1992). However, studies also show that individuals exercise less after they become parents (Bellows-Riecken and Rhodes 2008) and obesity risk increases with each additional child (Weng et al. 2004). We expect that parenthood-induced lifestyle changes will on average be more profound for biological parents than adoptive parents, as the latter are carefully pre-screened on health and health behaviours.

The aim of this study is to distinguish between the models described above by means of examining the relationship between parity and mortality for adoptive mothers and fathers, and mothers and fathers with biological children. The Swedish administrative registers provide information on the socioeconomic position of the parents in our study, meaning that we can also take account of the selection processes connecting socioeconomic status to completed parity. We can also examine whether the sex composition of the child group is associated with parental mortality, as this may be related to social support from children. Unfortunately, we have no variables that allow us to control for health and morbidity. However, our examination of adoptive parents who have undergone an extensive screening process will allow us to examine how parity is associated with mortality net of those factors. In general, our research design allows us to examine how having children is related to mortality, and to assess the extent to which this varies amongst those who have experienced pregnancies and those who have not. We can compare women with biological children to those with adopted children, where the latter have not borne the physiological costs, nor the potential benefits, of childbearing. We can also contrast this with the experience of fathers with biological and adopted children. Previous research has compared how the relationship between parity and mortality differs for men and women, but our study design allows us to compare men and women who have similar responsibilities in their role as parents, but where one group has not borne the physiological cost of childbearing.

In general, we argue that higher mortality for mothers with biological children, compared to adoptive mothers, would give support to physiological theoretical explanation models, particularly if mortality is increasing by parity. Similar patterns for men and women would overall be consistent with a larger role for social explanations focusing on parental depletion, and this would be true regardless if the children were biological or adopted. Large differences between adoptive parents and parents with biological children, in particular if these differences are similar by sex, would be consistent with a larger role for explanation by selection factors as well as lifestyle changes in response to parenthood. Furthermore, we can compare the mediating role of socioeconomic status according to whether adoptees were born in Sweden or abroad, and according to the gender of the children, to get a better insight into why parity might be associated with mortality. We also conduct analyses to examine the relationship between parity and cause-specific mortality for biological and adoptive parents. These cause-specific mortality analyses have the potential to shed light on the mechanisms for the relationship between parity and mortality for biological and adoptive parents. These analyses will shed light on the extent to which the relationship between parity and mortality can be explained by biomedical models, as well as health selection and lifestyle and behavioural changes induced by entry into parenthood. For example, mortality attributable to diseases of the circulatory system, or external causes such as accidents, allows us to speculate about lifestyle characteristics, or propensity to engage in risk-taking behaviours. Comparing the relationship before and after adjusting for socioeconomic status would give an approximation of whether this is largely related to socioeconomic factors, or selection on unobservable factors such as the underlying health of the parents.

## Data and Methods

### Data

In this study we use contemporary Swedish register data to analyse the relationship between parity and mortality for men and women. We look at birth cohorts born between 1915 and 1960 and link the population register to the Swedish mortality register, following them from 1975 to 2012. As we are studying the relationship between parity and mortality, it is necessary to study post-reproductive mortality so that the individuals under analysis will have reached completed fertility. Both men and women enter the analysis in 1975, or at age 45, whichever comes first. For the earliest cohort, born in 1915, this means that we are able to follow them up from age 60 to age 97, while for the latest cohort, born in 1960, we are able to follow them up from age 45 to age 52. This means that different birth cohorts contribute exposure to different ages in the analysis, though there is a substantial overlap.

Although Sweden has one of the highest rates of international adoption in the world (Selman 2002), this still means a low absolute number of adoptions relative to biological births in Sweden. This is compounded by looking at women and men who adopt and have no biological children of their own. We do not study parents of a mixed sibling group of both biological and adoptive children. As a result, we only study parents who have adopted up to four children. While our data do include parents who adopted five children, there is an insufficient number to produce reliable estimates for that category. Our final study size is 2,113,856 women experiencing 650,354 deaths, and 2,150,899 men experiencing 812,978 deaths. Descriptive information on the population used in the analyses is given in Tables 1 and 2.

**Table 1:** Descriptive statistics: biological and adoptive Swedish mothers, born 1915–1960

Variable	Category	Biological				Adoptive			
		*N*	%	Deaths	Rate	*N*	%	Deaths	Rate
Parity	0	275,353	13.2	112,945	15.1				
	1	365,313	17.5	137,839	13.1	17,428	58.8	6921	13.5
	2	816,570	39.2	203,017	9.1	11,175	37.7	1741	6.0
	3	423,389	20.3	110,299	9.5	911	3.1	108	4.8
	4	136,860	6.6	46,377	11.7	113	0.4	17	5.4
	5	43,057	2.1	18,512	14.3				
	6	15,260	0.7	7699	16.5				
	7	5955	0.3	3379	18.4				
	8	2472	0.1	1500	19.9				
Adoptee origin	Domestic					14,006	47.3	7766	16.4
	Transnational					15,168	51.2	960	2.8
	Mixed					453	1.5	61	4.7
Birth cohort	1915–1920	278,400	13.4	246,940	28.9	4407	14.9	3912	28.9
	1921–1925	231,004	11.1	156,780	19.1	3330	11.2	2184	18.2
	1926–1930	202,509	9.7	91,458	11.6	2735	9.2	1199	11.3
	1931–1935	185,438	8.9	53,120	7.6	2345	7.9	583	6.5
	1936–1940	198,297	9.5	34,895	5.3	2567	8.7	367	4.3
	1941–1945	257,465	12.4	28,577	4.0	3802	12.8	266	2.5
	1946–1950	268,124	12.9	17,548	2.9	4124	13.9	168	1.8
	1951–1955	234,548	11.3	8347	2.1	3481	11.8	74	1.2
	1956–1960	228,444	11.0	3902	1.5	2836	9.6	34	1.0
Education	Primary < 9 years	622,888	29.9	312,918	14.3	7262	24.5	3954	15.3
	Primary—9 years	217,972	10.5	43,961	7.9	2382	8.0	629	9.3
	Secondary—10–11 years	627,013	30.1	124,249	7.4	8213	27.7	1760	7.7
	Secondary—12 years	114,676	5.5	15,050	5.5	1788	6.0	215	4.8
	Tertiary—13–15 years	186,568	9.0	20,722	4.7	3585	12.1	362	4.2
	Tertiary—15+ years	197,535	9.5	21,452	4.2	4784	16.2	410	3.3
	Postgraduate	3830	0.2	513	4.7	86	0.3	11	4.6
	Missing	113,747	5.5	102,702	65.9	1527	5.2	1446	74.5
EGP	I	88,649	4.3	10,644	4.9	2274	7.7	212	3.8
	II	386,011	18.5	81,428	7.2	7596	25.6	1479	6.8
	III	271,277	13.0	42,488	6.2	4379	14.8	538	4.7
	IV	101,434	4.9	33,520	10.3	1453	4.9	516	11.0
	VI–VII	675,410	32.4	181,990	9.4	6874	23.2	1849	9.3
	Unknown	561,448	26.9	291,497	19.2	7051	23.8	4193	21.0

**Table 2:** Descriptive statistics: biological and adoptive Swedish fathers, born 1915–1960

Variable	Category	Biological				Adoptive			
		*N*	%	Deaths	Rate	*N*	%	Deaths	Rate
Parity	0	418,005	19.72	193,361	19.3				
	1	342,262	16.14	151,370	17.0	18,804	60.75	8505	16.2
	2	760,393	35.87	240,620	12.2	11,045	35.68	2282	8.0
	3	399,495	18.84	130,379	12.6	960	3.1	144	5.9
	4	134,150	6.33	53,137	15.1	145	0.47	35	8.8
	5	42,950	2.03	20,252	17.8				
	6	14,895	0.7	8124	20.3				
	7	5574	0.26	3351	22.3				
	8	2221	0.1	1418	23.6				
Adoptee origin	Domestic					15,777	50.97	9410	19.6
	Transnational					14,656	47.35	1456	4.2
	Mixed					521	1.68	100	6.7
Birth cohort	1915–1920	273,628	12.91	261,040	37.8	4811	15.54	4534	36.7
	1921–1925	229,607	10.83	190,018	27.0	3521	11.37	2711	24.0
	1926–1930	202,019	9.53	127,590	18.0	3029	9.79	1621	14.4
	1931–1935	187,313	8.84	81,076	12.3	2645	8.54	856	8.7
	1936–1940	201,422	9.5	53,891	8.4	2859	9.24	523	5.6
	1941–1945	266,330	12.56	43,052	6.0	4166	13.46	438	3.8
	1946–1950	277,524	13.09	26,497	4.3	4366	14.1	196	2.0
	1951–1955	243,948	11.51	12,881	3.1	3250	10.5	65	1.2
	1956–1960	238,154	11.23	5967	2.2	2307	7.45	22	0.8
Education	Primary < 9 years	614,743	29	333,590	16.8	7709	24.9	4074	15.9
	Primary—9 years	193,070	9.11	33,472	8.4	2000	6.46	367	7.7
	Secondary—10–11 years	477,555	22.53	118,979	10.3	6080	19.64	1605	9.9
	Secondary—12 years	262,291	12.37	67,712	9.5	4757	15.37	1202	8.7
	Tertiary—13–15 years	160,351	7.56	28,662	7.4	3061	9.89	539	6.9
	Tertiary—15+ years	199,064	9.39	34,477	6.9	4439	14.34	691	6.0
	Postgraduate	17,247	0.81	3393	6.9	407	1.31	71	6.0
	Missing	195,624	9.23	181,727	75.7	2501	8.08	2417	84.5
EGP	I	173,623	8.19	27,021	6.4	3894	12.58	527	5.4
	II	332,028	15.66	81,199	9.1	6501	21	1410	7.8
	III	173,705	8.19	50,977	11.2	2830	9.14	803	10.3
	IV	87,488	4.13	7890	4.2	1112	3.59	60	2.4
	VI–VII	913,977	43.11	328,083	13.4	10,477	33.85	3415	11.2
	Unknown	439,124	20.71	306,842	29.9	6140	19.84	4751	31.0

### Covariates

We estimate two models to examine the main effects of parity amongst biological and adoptive parents. In the first model we adjust only for the birth cohort of our index individuals. In the second model, we also adjust for socioeconomic status, completed educational attainment, and whether adoptive parents raised a domestically adopted child, a transnational adoptee, or a sibling group with a mix of both. The control variable for socioeconomic status is based upon the Erikson, Goldthorpe and Portocarero occupational class scheme (EGP) (Erikson et al. 1979), measured between ages 30 and 40 using information on occupation from the Swedish censuses in 1960, 1970, 1980, and 1990. The EGP variable used in this study is divided into the following categories: upper service class, including self-employed professionals (EGP = I); lower service class (EGP = II); routine non-manual (EGP = III); self-employed non-professionals, farmers, and fishermen (EGP = IV); skilled and unskilled workers (EGP = VI–VII); and unknown/other.

The control variable for education consists of seven categories. Due to the wide range of birth cohorts that we study, the Swedish educational system underwent some changes during that period. For that reason we describe the categories used for education by both the ‘level’, as well as the number of years that would typically be need to attain that level. The eight categories of the educational attainment variable are primary (< 9 years), primary (9 years), secondary (10–11 years), secondary (12 years), tertiary (13–15 years), tertiary, but not including postgraduate qualifications (15+ years), and postgraduate qualifications (approximately 16–20 years). The final, eighth, category indicates whether the variable for education has a missing value. The motivation for the inclusion of the educational attainment and attained socioeconomic status variables is to adjust for the fact that adoptive parents undergo a screening process that biological parents are not subject to, as well as the fact that there are numerous education- and socioeconomic status-related selection processes operating in regard to the fertility behaviour of biological mothers and fathers.

### Statistical Analyses

To study the relationship between parity and mortality we use survival analysis. The general proportional hazards model is expressed as:

$$h(t | X_{1} , \ldots , X_{k} ) = h_{0} \left( t \right) \exp \left( {\mathop \sum \limits_{j = 1}^{k} \beta_{j} X_{j} \left( t \right)} \right),$$

where $h\left( {t | X_{1} , \ldots , X_{k} } \right)$ is the hazard rate for individuals with characteristics $X_{1} , \ldots , X_{k}$ at time *t*, *h*_*0*_ is the baseline hazard at time *t*, and *β*_*j*_, *j* = 1,…, *k* are the estimated coefficients. Since the failure event in our analysis is the death of the individual, the baseline hazard of our model *h*_0_ is age, measured as time since age 45 for those born after 1930, or the age of the index person in 1975 for those born before 1930. It is assumed to follow a Gompertz distribution, defined as:

$$h_{0} = \exp \left( {\gamma t} \right)\exp \left( {\beta_{0} } \right),$$

where *γ* and *β*_0_ are ancillary parameters that control the shape of the baseline hazard. The Gompertz distribution is a continuous probability distribution that has an exponentially increasing failure rate, and closely approximates the hazard of mortality in human adults. We conduct separate analyses for men and women. To provide a clear indicator of what these differences in the hazard of mortality mean, we have conducted additional analyses to translate the estimated hazard ratios from the Gompertz models into differences in life expectancy for adoptive parents and parents of biological children. Life expectancy was calculated by using Swedish life tables by sex from 2007 assuming that every per cent increase in hazard of mortality, relative to our reference group, translated into a similar increase in age-specific mortality rates after age 45.

In addition to our main analyses, we also split the group of adoptive parents into those who adopted children domestically, transnationally, or a combination of both and compare the mortality of these different groups to biological parents and childless individuals. We also examine whether there are interactions between parental educational level and parity for biological and adoptive parents, and whether the sex composition of the child group is associated with mortality for biological and adoptive parents. For these interaction analyses we split educational level into three categories: less than senior high school (less than 9 years), senior high school (10–12 years), and any tertiary education (13+ years). For the sex composition of children we distinguish between groups that are all girls, all boys, or mixed. In further additional analyses we assess whether the relationship between parity and mortality differs by cause of death, censoring for other causes of death. The causes we focus upon are mortality attributable to neoplasms, diseases of the circulatory system, external causes attributable to accidents, suicides, and events of undetermined intent, and all other causes. These cause-specific outcome variables were coded using the WHO’s International Classification of Diseases (ICD), versions 8, 9, and 10, taking into account the transition between these versions in 1996 in Sweden (Janssen and Kunst 2004). As we can no longer assume independent right-censoring as our causes of death are dependent upon each other we can no longer estimate the marginal effect (the effect of our covariates on a specific cause of death in the absence of other causes of deaths). We can, however, still examine the extent to which parity mediates mortality for different causes of death.

## Results

### Multivariate Analyses

**Fig. 1:**
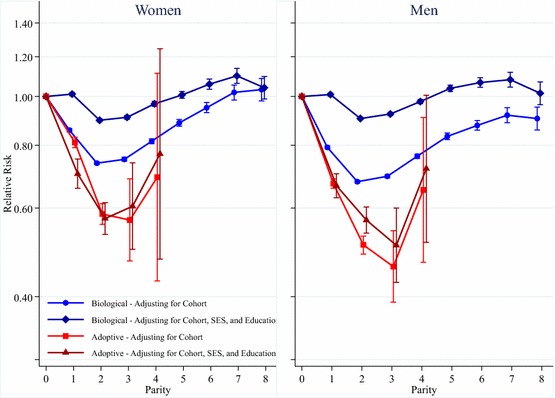
Parity and mortality for Swedish women and men, birth cohorts 1915–1960

Figure 1 shows the results for the relationship between parity and mortality for women and men, with the results for women in the left panel and the results for men in the right panel. Please note that the *y*-axis is plotted on a log scale. These analyses include biological and adoptive parents simultaneously, where childless individuals are a common reference category. Both panels in Fig. 1 present the results from two models, the first without adjustment for socioeconomic status and educational attainment and the latter including adjustment for these covariates. The full results tables upon which these graphs are based can be found in the appendices, in Tables S1 and S2. The results in the left panel of Fig. 1 show these results for women. As can be seen, the mortality of adoptive mothers is always lower than that of mothers with biological children. After adjusting for SES and educational attainment, the estimates for adoptive mothers do not vary a great deal, indicating that these types of parents were already a select group in terms of socioeconomic status and related lifestyle factors thanks to the vetting procedure conducted by the adoption authorities in Sweden. The results for adoptive mothers show that they have much lower mortality than childless women. Mothers with two adopted children have lower mortality than those with one adopted child. Mothers with three adopted children have a similar hazard of mortality to those with two adopted children, though the hazard is higher for mothers with four adopted children. In the latter case the confidence intervals are very wide, reflecting the large standard error based upon the relatively small number of mothers with so many adopted children. Overall, the results for men, shown in the right panel of Fig. 1, are very similar to those for women.

The results for mothers with biological children, however, change substantially after adjusting for SES and educational attainment. Figure 1 shows that, when only adjusting for birth cohort, the mortality of women with six children or fewer is lower than that of childless women. The mortality of women with seven or eight children is slightly higher than that of childless women, but the differences are small and not statistically significant. Mothers with two biological children have the lowest mortality, while it is higher for those with one child, and mortality increases with parity for mothers with three children or more. After adjusting for SES and educational attainment, the differences in mortality by parity for mothers with biological children are substantially less pronounced, particularly in comparison with childless women. Mothers with two or three children have the lowest mortality, but mothers with one child have the same mortality as the childless. Mothers with six or more children have higher mortality than the childless. Figure 1 shows that the results for fathers with biological children are similar to those for mothers with biological children. In the model where we adjust for cohort but not for SES and educational attainment, fathers always have lower mortality than childless men, but men with two children have the lowest mortality. The U-shaped nature of the relationship with parity is similar to that seen for women. After adjusting for SES and educational attainment, men with two children still have the lowest mortality compared to all others, while men with one, or five or more, children have higher mortality than the childless.

We have conducted additional calculations to translate the differences in the hazards of mortality for adoptive parents and parents of biological children into estimates for differences in life expectancy, based upon the fully adjusted model in Fig. 1. The results from these calculations for remaining life expectancy at age 45, shown in Figure S1 in the appendices, are based on mortality conditions in Sweden in 2007. Based upon this 2007 life table, we find that, compared to childless women, women who have adopted one child have a life expectancy that is greater by over 3 years, while it is greater by 5 years for adoptive mothers with two or three children. Other estimates for relative differences in remaining life expectancy are shown in Figure S1.

**Fig. 2:**
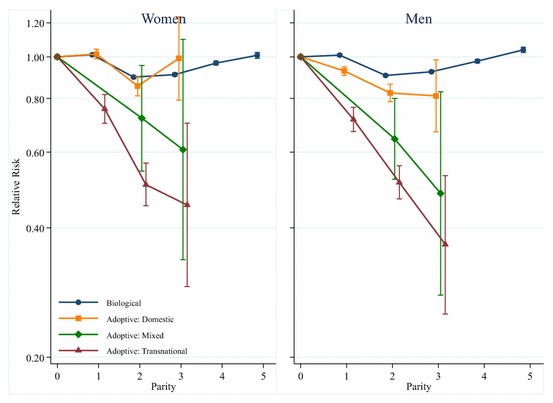
Parity and mortality for Swedish women and men, birth cohorts 1915–1960, by adoptee region of origin

To investigate the mortality of adoptive parents in greater detail we conducted analyses where we examined how the hazard varies according to whether the adopted children were adopted domestically or transnationally. These estimates from these models are shown in Fig. 2, with the results for women in the left panel and the results for men in the right panel. These estimates are based upon models that include control variables for cohort, socioeconomic status, and educational attainment. We look at adoptive parents who adopted domestically, who adopted transnationally, and who had a mix of domestic and transnational adoptees. A plot of the results for parents with biological children is included for comparison’s sake. As shown in Fig. 2, the mortality of adoptive mothers who adopted children domestically is almost identical to that of mothers with biological children, while the mortality of women who adopted children transnationally is much lower. The results for adoptive mothers who have a mix of domestic and international adoptees show that mortality amongst that group is lower than amongst the mothers of domestic adoptees, but higher than the mothers of transnational adoptees. However, the confidence intervals overlap with the estimates for domestic and transnational adoptive mothers, and additional Wald tests show that there is no statistically significant difference between these estimated coefficients. Overall, the results for men are very similar to those seen for women, though fathers with domestically adopted children have slightly lower mortality than fathers with biological children.

**Fig. 3:**
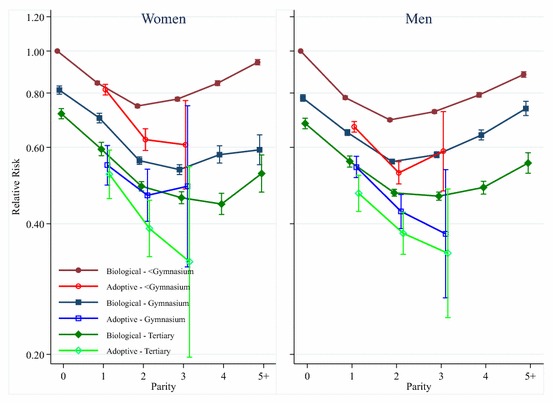
Parity and mortality for Swedish women and men, birth cohorts 1915–1960, by parental educational level

In further analyses we examine whether the association between parity and mortality varies according to the educational attainment of the mother and father, and whether this varies for biological and adoptive parents. We cannot examine adoptive parents with four children, as there are too few to divide them by educational level. The results from those interactions are shown in Fig. 3, with the results for women in the left panel and the results for men in the right panel. The results in each of the two panels are based on one statistical model, interacting parent type (biological vs. adoptive) with educational attainment and parity, with childless biological parents with less than a senior high school level education as the reference category. The left-hand panel of Fig. 3 shows that there are differences in the parity–mortality relationship by maternal educational level. Amongst biological mothers, those who have higher levels of education have lower mortality at each level of parity. It can be seen that amongst those with less than a senior high school level education, there is a clear U-shaped association between parity and mortality, where women who have two children have the lowest mortality. Amongst women with a senior high school level education, those who have three children have the lowest mortality, and mortality increases marginally amongst those who have four, or five or more, children. Amongst biological mothers with a tertiary education, those who have four children have the lowest mortality. Amongst adoptive mothers we also see that higher levels of education are associated with lower mortality, but in this case having more adoptive children is generally associated with having lower mortality.

The right panel of Fig. 3 shows the results from interaction analyses between parent type, educational level, and parity for men. These results are similar to those seen for women, but amongst biological fathers at all education levels having four or five children is clearly associated with an increase in mortality. Mortality is lowest amongst biological fathers with two children except for biological fathers who have a tertiary level of education, amongst whom those with two or three children have the lowest mortality. Amongst adoptive fathers, those with higher levels of education have lower mortality, while having more adopted children is associated with lower mortality, with the exception of adoptive fathers with less than senior high school level education who have three adopted children.

**Fig. 4:**
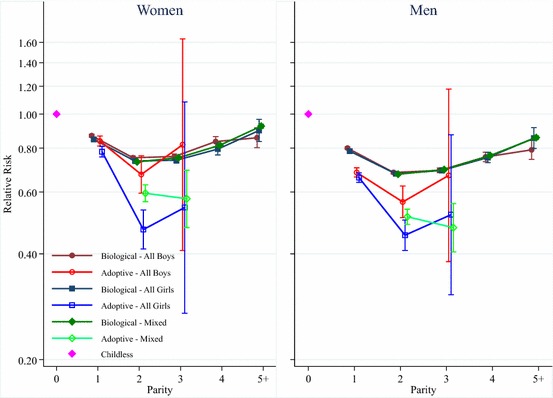
Parity and mortality for Swedish women and men, birth cohorts 1915–1960, by sex composition of child group

The results from additional analyses examining how the sex composition of the child group is related to the mortality of the parents are shown in Fig. 4. The reference category is childless parents, and the results in the left and right panels are both based on one model interaction parent type with the sex composition of the children and parity. The left panel shows the results for women, and the right panel the results for men. The estimates for men and women show that amongst biological parents there are no significant differences in survival between those who have all boys, all girls, or a mixed sex group of children. Amongst adoptive parents with one child, there are no differences in mortality depending on whether they adopted a boy or a girl. However, amongst adoptive parents with two children, those who adopted girls have lower mortality than those who adopted boys, and the difference is statistically significant. Amongst those who adopted three children the differences in mortality level cannot be statistically distinguished, but the point estimates indicate that those who adopted all boys have higher mortality.

**Fig. 5:**
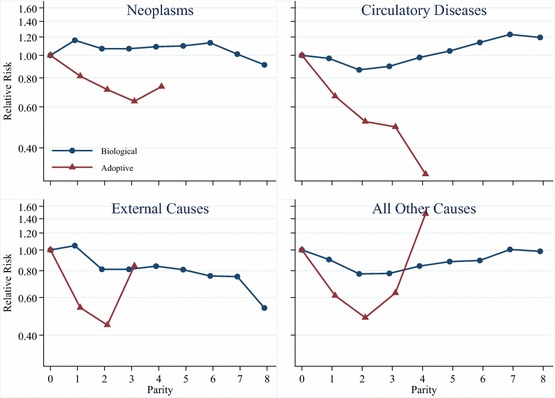
Parity and cause-specific mortality for Swedish women, birth cohorts 1915–1960

**Fig. 6:**
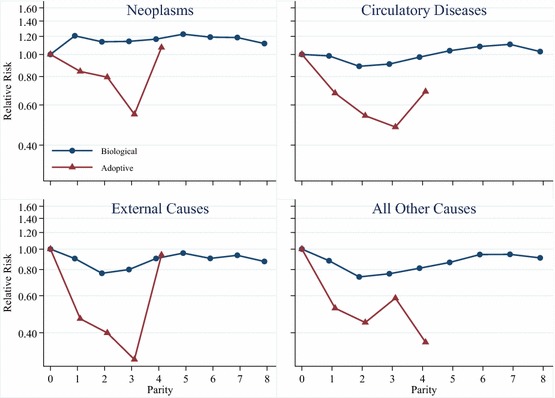
Parity and cause-specific mortality for Swedish men, birth cohorts 1915–1960

In a set of additional analyses we examine the relationship between parity and mortality by cause of death. These results are shown in Fig. 5 for women and in Fig. 6 for men. We exclude the confidence intervals from Figs. 5 and 6 because they are wide and make it much more difficult to read the results. However, the full results tables upon which these graphs are based, including standard errors and confidence intervals, are given in Appendices in Tables S3 and S4. Each of the four panels in Figs. 5 and 6 shows the results from separate models interacting parent type with parity, and adjusting for birth year, attained socioeconomic status, and educational attainment. Figures 5 and 6 show that the results for mortality attributable to diseases of the circulatory system are similar to all-cause mortality for both mothers and fathers of biological children at lower parities. The results for mortality attributable to neoplasms, however, show that parous women and men have higher mortality than childless women. Additional analyses, not shown, where we study the relationship between parity and breast cancer for women show that women with children have lower mortality attributable to breast cancer than childless women, consistent with previous research (Grundy and Kravdal 2010). This means that the higher hazard of neoplasm-attributable mortality amongst parous women is driven by cancers other than breast cancer. The results in Figs. 5 and 6 also show that mothers and fathers of biological children have lower mortality from external causes attributable to accidents, suicides, and events of undetermined intent, as well as from all other causes. The results from models examining cause-specific mortality for adoptive parents show that cause-specific mortality for all the causes we study is lower for adoptive parents than it is for childless women and men. The results for mortality attributable to neoplasms, diseases of the circulatory system, external causes, and all other causes all show lower mortality for adoptive mothers and fathers with two adopted children compared to those with one adopted child, though the lower hazard of mortality from external causes is particularly pronounced. The results for mortality attributable to neoplasms and diseases of the circulatory system show that adoptive mothers and fathers with three adopted children have lower mortality than those with two adopted children. However, we encourage caution in the interpretation of the results for adoptive parents with three or four adopted children, as there are few deaths in these categories, particularly when divided amongst specific causes of death.

## Discussion

In comparing biological and adoptive mothers and fathers we have attempted to distinguish between different potential mechanisms for the relationship between parity and mortality. It should be noted that there is no good reason to suspect that one theory fully explains the parity–mortality association, but in this study we have tried to evaluate the relative importance of different explanatory mechanisms. First, we do find support for biomedical explanations for the relationship between parity and mortality. Our analyses corroborate previous research that shows that childless women have the highest risk of breast cancer and having more children reduces the risk further. Previous research using Swedish and Norwegian register data has also shown that parity is associated with the risk of cervical and ovarian cancer (Grundy and Kravdal 2010; Barclay et al. 2016). These findings are consistent with theoretical mechanisms that describe the importance of exposure to ovarian hormones such as oestrogen and progesterone for cancer development (Kelsey et al. 1993). Nevertheless, we know that biomedical models do not fully explain the parity–mortality association, as parity is associated with mortality for men as well as for adoptive men and women without biological children.

In our study we do not find any clear evidence for the disposable soma theory (Kirkwood and Holliday 1979), which describes a direct trade-off between childbearing and longevity. The disposable soma theory implies that there should be a positive association between parity and mortality, with childless individuals having the lowest mortality net of other factors. Furthermore, the burden of childbearing should be far more substantial for women, which we do not observe. It is possible that disposable soma theory was more relevant for explaining the relationship between parity and mortality in historical settings with higher fertility and without the social welfare state and advanced public health-promoting infrastructure of the modern Swedish context. The evidence for this is mixed. A systematic review of studies on the relationship between parity and mortality using historical data has shown that the results across different studies are inconsistent, as some reveal a positive relationship between parity and longevity, while others show a negative relationship (Hurt et al. 2006). Some studies using data from historical and less developed societies show no clear relationship between parity and mortality for men, but others do (Friedlander 1996; Smith et al. 2002; Hurt et al. 2004). Overall, disposable soma theory appears to have less explanatory power than the other explanations proposed to account for the parity–mortality association.

We also considered whether depletion factors of a social nature might explain the relationship between parity and mortality. Social depletion involves the stress and various costs involved in raising children, including lost earnings from reduced labour force participation, and would be likely to apply to both men and women, and to both biological and adoptive parents. In general, a positive association between parity and mortality would be consistent with a social depletion explanation. Overall, our results do not support social depletion as a dominant explanation for the parity–mortality association, though we do find that mothers and fathers who have five or six or more children have higher mortality than childless individuals even after adjusting for social class and education. However, since less than 4% of men and women in Sweden have five or more children, we suspect that there may be selection mechanisms that account for this association that are not captured by our control variables.

Indeed, we suspect that selection processes largely explain the curvilinear relationship between parity and mortality amongst biological parents in contemporary populations. The similar results for men and women, as well as the curvilinear nature of the relationship for biological mothers and fathers, suggest that selection on socioeconomic status or health drives the parity–mortality association, and particularly at higher parities. The fact that the results for the relationship between parity and mortality change substantially after adjusting for socioeconomic status and educational attainment suggests that socioeconomic status-related selection processes play an important role in explaining this relationship. However, the fact that the curvilinear pattern remains even after adjusting for socioeconomic status suggests that other selection factors may also play an important role. Childless individuals are likely to be negatively selected out of partnering and parenthood, while those who have many children, particularly amongst groups with lower levels of education, may be drawn from groups who are more reckless in terms of health habits as well as behaviours such as driving. The lower mortality of adoptive parents provides additional evidence that selection on health behaviours is an important cause of lower mortality. However, our analyses of adoptive parents do allow us to examine how number of children is related to mortality after a careful selection on parents with good health, positive health behaviours, a supportive relationship and social network, and a stable occupation with more than adequate financial resources. Although this does not allow us to get away from selection, it is at least selection based on criteria related to a documented screening process rather than selection processes that we have to infer without solid evidence. Amongst these parents who adopt transnationally we see that more children are associated with a decrease in mortality. This suggests that the presence of children does do something positive to increase health or health behaviours. Adopting children in Sweden is time-consuming and expensive, but not to the extent that parental wealth would drive such clear mortality differences amongst parents who adopt three children versus two, for example. The lower mortality pattern of parents with several adoptive children also remains after we control for socioeconomic covariates.

If the presence of children does improve health or health behaviours, it is likely to be attributable either to social support from children, or to lifestyle changes induced by entry into parenthood. Given previous research on this topic (e.g. Chilcoat and Breslau 1996; Nock 1998; McDermott et al. 2006), we suspect that lifestyle changes probably do play an important role in the health advantage observed amongst those who have two or three children relative to the childless, particularly for biological parents. This is also suggested by our cause-of-death-specific analyses that show that deaths attributable to external causes such as accidents are lower amongst parents than amongst the childless for both men and women. We also observe lower mortality from circulatory diseases amongst biological parents at the most common parities, which is likely to be related to lifestyle and behaviour, though the higher overall mortality from neoplasms means that those conclusions cannot be definitive. Given the screening process operated by the Swedish adoption authorities, we did not expect that entry into parenthood would induce major changes in health behaviours amongst those who adopted children transnationally, but we did observe that the hazard of mortality from external causes was particularly low for adoptive mothers and fathers. While these individuals were selected on already having health behaviours that would be conducive to raising children in a positive environment, the domesticating effect of childrearing may have induced even further increases in sober and responsible behaviour.

Another potential explanation for why adoptive parents with more children have lower mortality is the social support that children can provide for parents in their post-reproductive years. Studies have shown that the attained socioeconomic status of children is related to parental mortality (Torssander 2013; Friedman and Mare 2014; Zimmer et al. 2016) and there are consistent gender differences in patterns of caregiving (Rossi and Rossi 1990). We do find that adoptive parents who adopt two girls have lower mortality than adoptive parents who have two boys, though there are no significant differences amongst parents who adopt one, or three, children. Since survival by the gender of the children is not consistent across family size, and since adoptive parents may have been able to exercise a preference for the sex of the adopted child in the past, we suspect that the difference in mortality between parents who adopt two boys versus those who adopt two girls could potentially be the product of selection processes related to parental preferences of child characteristics. Our analyses of biological parents based on the sex composition of the children do not find any differences by the gender composition of the child group. Furthermore, mortality decreases up to two children, before increasing again, which is contrary to social support as a dominant explanation for the parity–mortality association.

A potentially important factor that we did not take into consideration in our study was parental age at the time of birth and the time of adoption. Part of the reason for this was that a key comparison group in our analyses was childless individuals, who do not have an age at birth. Parental age at birth is associated with changes to socioeconomic status, reproductive ageing, as well as important period changes (Barclay and Myrskylä 2016). However, studies that have examined the parity–mortality relationship while adjusting for age at first birth have found that it makes little difference to the overall estimated parity–mortality pattern (e.g. see Barclay et al. 2016). Furthermore, parental age at the time of adoption is regulated in Sweden, meaning that if either parent is below 25, or above 43, they are essentially unable to adopt children. Since adoptive parents do not have to contend with ageing of the reproductive system and are already selected on having comfortable socioeconomic conditions, it is unlikely that parental age at the time of adoption would play an important role in the mortality of the parents.

Overall, the explanation for the association between parity and mortality remains unclear. Biomedical factors may be a part of the explanation for women, and lifestyle changes induced by entry into parenthood are likely to be important for both men and women. Social support may play a role, but the evidence for that factor is not strong. Selection processes related to socioeconomic status and health are likely to explain a large part of the association between parity and mortality for biological parents. In particular, for childless individuals (and to a lesser extent parents with one child) we suspect that negative behaviours and traits associated with both childlessness and health are responsible for the elevated mortality that we observe. However, we also observe lower mortality with increasing parity even amongst adoptive parents who are selected on documented criteria related to socioeconomic status and health. Future research might attempt to further explore the explanations underlying the parity–mortality association by investigating other factors that we were unable to examine here, such as the potential for genetics or personality factors to account for the relationship.

## Electronic supplementary material

Below is the link to the electronic supplementary material.

Supplementary material 1 (PDF 2270 kb)
