# Supplementary material for: Parity and Mortality: An Examination of Different Explanatory Mechanisms Using Data on Biological and Adoptive Parents
Source: Eur J Popul. 2018 Feb 21;35(1):63–85. doi: 10.1007/s10680-018-9469-1 (PMC6357259; doi:10.1007/s10680-018-9469-1)
Supplement: Supplementary file 1 — Supplementary material 1 (PDF 2270 kb) [file 10680_2018_9469_MOESM1_ESM.pdf]

Appendices

Table S1. Results: All-cause Mortality for Swedish Women, Born 1915 to 1960.

Variable	Category	RR	SE	95% CI	RR	SE	95% CI
Parity	Childless	1.00			1.00		
	Biological - 1	0.86	0.00	0.85-0.86	1.01	0.00	1.00-1.02
	Biological - 2	0.74	0.00	0.73-0.74	0.90	0.00	0.89-0.90
	Biological - 3	0.75	0.00	0.74-0.76	0.91	0.00	0.90-0.92
	Biological - 4	0.82	0.00	0.81-0.82	0.97	0.01	0.96-0.98
	Biological - 5	0.89	0.01	0.87-0.90	1.01	0.01	0.99-1.02
	Biological - 6	0.95	0.01	0.93-0.97	1.06	0.01	1.03-1.08
	Biological - 7	1.02	0.02	0.98-1.05	1.10	0.02	1.06-1.14
	Biological - 8	1.03	0.03	0.98-1.09	1.04	0.03	0.99-1.10
	Adoptive - 1	0.81	0.01	0.79-0.83	0.70	0.02	0.66-0.75
	Adoptive - 2	0.58	0.01	0.56-0.61	0.57	0.02	0.53-0.62
	Adoptive - 3	0.57	0.05	0.47-0.69	0.61	0.06	0.50-0.74
	Adoptive - 4	0.69	0.17	0.43-1.11	0.77	0.19	0.48-1.24
Birth Cohort	1915-1920	1.00			1.00		
	1921-1925	0.91	0.00	0.91-0.92	1.06	0.01	1.05-1.08
	1926-1930	0.82	0.00	0.82-0.83	1.12	0.01	1.11-1.14
	1931-1935	0.78	0.00	0.77-0.79	1.14	0.01	1.12-1.16
	1936-1940	0.77	0.00	0.76-0.78	1.21	0.01	1.19-1.23
	1941-1945	0.81	0.01	0.80-0.82	1.38	0.01	1.35-1.40
	1946-1950	0.80	0.01	0.78-0.81	1.45	0.01	1.42-1.47
	1951-1955	0.79	0.01	0.77-0.81	1.53	0.02	1.49-1.57
	1955-1960	0.77	0.01	0.74-0.79	1.52	0.03	1.47-1.57
Origin of Adoptees	Domestic				1.46	0.05	1.36-1.56
	Transnational				1.00		
	Mixed				1.16	0.16	0.89-1.52
Education	Primary $<$ 9 years				1.00		
	Primary - 9 years				0.91	0.00	0.91-0.92
	Secondary - 10-11 years				0.85	0.00	0.84-0.85
	Secondary - 12 years				0.80	0.01	0.78-0.81
	Tertiary - 13-15 years				0.71	0.01	0.70-0.72
	Tertiary - 15+ years				0.65	0.00	0.64-0.66
	Post-graduate				0.61	0.03	0.56-0.67
	Missing				7.51	0.03	7.45-7.57
EGP	I				1.00		0.00-0.00
	II				0.99	0.01	0.97-1.01
	III				1.08	0.01	1.06-1.10
	IV				0.99	0.01	0.96-1.01
	VI-VII				1.14	0.01	1.12-1.17
	Unknown				1.09	0.01	1.07-1.12

Table S2. Results: All-cause Mortality for Swedish Men, Born 1915 to 1960.

Variable	Category	RR	SE	95% CI	RR	SE	95% CI
Parity	Childless	1.00			1.00		
	Biological - 1	0.79	0.00	0.79-0.80	1.01	0.00	1.00-1.02
	Biological - 2	0.68	0.00	0.67-0.68	0.90	0.00	0.90-0.91
	Biological - 3	0.69	0.00	0.69-0.70	0.92	0.00	0.92-0.93
	Biological - 4	0.76	0.00	0.75-0.77	0.98	0.00	0.97-0.99
	Biological - 5	0.83	0.01	0.82-0.85	1.04	0.01	1.02-1.05
	Biological - 6	0.88	0.01	0.86-0.90	1.07	0.01	1.04-1.09
	Biological - 7	0.92	0.02	0.89-0.95	1.08	0.02	1.04-1.12
	Biological - 8	0.90	0.02	0.86-0.95	1.01	0.03	0.96-1.07
	Adoptive - 1	0.67	0.01	0.66-0.69	0.66	0.02	0.63-0.70
	Adoptive - 2	0.51	0.01	0.49-0.53	0.57	0.02	0.54-0.60
	Adoptive - 3	0.46	0.04	0.39-0.54	0.51	0.04	0.43-0.60
	Adoptive - 4	0.65	0.11	0.47-0.91	0.72	0.12	0.51-1.01
Birth Cohort	1915-1920	1.00			1.00		
	1921-1925	0.90	0.00	0.89-0.90	0.98	0.01	0.97-0.99
	1926-1930	0.80	0.00	0.80-0.81	1.07	0.01	1.06-1.09
	1931-1935	0.72	0.00	0.71-0.72	1.08	0.01	1.07-1.09
	1936-1940	0.63	0.00	0.62-0.64	1.06	0.01	1.05-1.07
	1941-1945	0.58	0.00	0.57-0.58	1.12	0.01	1.10-1.14
	1946-1950	0.53	0.00	0.52-0.53	1.10	0.01	1.09-1.12
	1951-1955	0.49	0.00	0.48-0.50	1.13	0.01	1.11-1.15
	1955-1960	0.45	0.01	0.44-0.46	1.06	0.02	1.03-1.09
Origin of Adoptees	Domestic				1.41	0.04	1.33-1.49
	Transnational				1.00		
	Mixed				1.08	0.11	0.88-1.33
Education	Primary < 9 years				1.00		
	Primary - 9 years				1.04	0.01	1.03-1.05
	Secondary - 10-11 years				0.93	0.00	0.92-0.93
	Secondary - 12 years				0.83	0.00	0.82-0.84
	Tertiary - 13-15 years				0.77	0.00	0.76-0.78
	Tertiary - 15+ years				0.71	0.00	0.70-0.72
	Post-graduate				0.63	0.01	0.61-0.66
	Missing				8.06	0.03	8.01-8.12
EGP	I				1.00		
	II				1.05	0.01	1.04-1.07
	III				1.12	0.01	1.10-1.14
	IV				0.95	0.01	0.93-0.98
	VI-VII				1.19	0.01	1.17-1.20
	Unknown				0.99	0.01	0.98-1.01

Table S3. Results: Cause-specific Mortality for Swedish Women, Born 1915 to 1960. Model 1 adjusts for birth cohort. Model 2 adjusts for birth cohort, educational attainment, adult socioeconomic status, and origin of adopted children.

		Model 1			Model 2		
Cause-of-death	Parity	RR	SE	95% CI	RR	SE	95% CI
Neoplasms	Childless	1.00			1.00		
	Biological - 1	0.93	0.01	0.91-0.94	1.16	0.01	1.14-1.18
	Biological - 2	0.82	0.01	0.81-0.83	1.07	0.01	1.05-1.08
	Biological - 3	0.82	0.01	0.81-0.83	1.07	0.01	1.05-1.09
	Biological - 4	0.85	0.01	0.83-0.86	1.09	0.01	1.07-1.11
	Biological - 5	0.89	0.01	0.87-0.92	1.10	0.02	1.07-1.13
	Biological - 6	0.93	0.02	0.89-0.98	1.13	0.03	1.08-1.18
	Biological - 7	0.88	0.03	0.82-0.94	1.01	0.04	0.94-1.09
	Biological - 8	0.86	0.05	0.77-0.96	0.91	0.05	0.82-1.01
	Adoptive - 1	0.87	0.02	0.83-0.91	0.81	0.04	0.74-0.90
	Adoptive - 2	0.66	0.03	0.61-0.72	0.71	0.04	0.64-0.79
	Adoptive - 3	0.55	0.09	0.40-0.76	0.63	0.11	0.46-0.88
	Adoptive - 4	0.62	0.25	0.28-1.38	0.73	0.30	0.33-1.65
Diseases of the circulatory system	Childless	1.00			1.00		
	Biological - 1	0.86	0.01	0.85-0.87	0.97	0.01	0.96-0.98
	Biological - 2	0.75	0.00	0.74-0.75	0.87	0.01	0.86-0.88
	Biological - 3	0.78	0.01	0.77-0.79	0.90	0.01	0.89-0.91
	Biological - 4	0.87	0.01	0.85-0.88	0.98	0.01	0.96-1.00
	Biological - 5	0.97	0.01	0.95-0.99	1.04	0.01	1.02-1.07
	Biological - 6	1.08	0.02	1.04-1.12	1.14	0.02	1.10-1.18
	Biological - 7	1.20	0.03	1.14-1.26	1.23	0.03	1.17-1.29
	Biological - 8	1.23	0.05	1.14-1.32	1.19	0.04	1.11-1.28
	Adoptive - 1	0.83	0.02	0.80-0.87	0.67	0.04	0.59-0.76
	Adoptive - 2	0.58	0.02	0.53-0.62	0.52	0.04	0.45-0.60
	Adoptive - 3	0.50	0.09	0.35-0.71	0.49	0.09	0.34-0.72
	Adoptive - 4	0.29	0.20	0.07-1.14	0.31	0.22	0.08-1.24
External causes	Childless	1.00			1.00		
	Biological - 1	0.80	0.02	0.76-0.83	1.05	0.02	1.01-1.09
	Biological - 2	0.59	0.01	0.57-0.61	0.81	0.02	0.78-0.84
	Biological - 3	0.59	0.01	0.57-0.62	0.81	0.02	0.78-0.85
	Biological - 4	0.63	0.02	0.59-0.66	0.84	0.03	0.79-0.89
	Biological - 5	0.63	0.03	0.58-0.69	0.81	0.04	0.74-0.88
	Biological - 6	0.60	0.04	0.52-0.70	0.76	0.06	0.65-0.87
	Biological - 7	0.63	0.07	0.51-0.78	0.75	0.08	0.60-0.93
	Biological - 8	0.49	0.09	0.34-0.72	0.54	0.10	0.37-0.78
	Adoptive - 1	0.65	0.04	0.57-0.75	0.54	0.09	0.39-0.74
	Adoptive - 2	0.45	0.06	0.35-0.57	0.45	0.08	0.32-0.62
	Adoptive - 3	0.79	0.28	0.39-1.57	0.84	0.32	0.40-1.75
	Adoptive - 4	0.00	0.00	.	0.00	0.00	.
All other causes	Childless	1.00			1.00		
	Biological - 1	0.79	0.01	0.78-0.80	0.90	0.01	0.89-0.92
	Biological - 2	0.66	0.00	0.65-0.67	0.77	0.01	0.76-0.78
	Biological - 3	0.67	0.01	0.65-0.68	0.78	0.01	0.76-0.79
	Biological - 4	0.74	0.01	0.72-0.75	0.84	0.01	0.82-0.86
	Biological - 5	0.80	0.01	0.78-0.83	0.88	0.01	0.85-0.91
	Biological - 6	0.83	0.02	0.79-0.87	0.89	0.02	0.85-0.94
	Biological - 7	0.96	0.03	0.89-1.02	1.01	0.03	0.94-1.08
	Biological - 8	0.98	0.05	0.89-1.09	0.99	0.05	0.89-1.09
	Adoptive - 1	0.74	0.02	0.71-0.78	0.61	0.04	0.53-0.70
	Adoptive - 2	0.52	0.03	0.47-0.57	0.48	0.04	0.41-0.56
	Adoptive - 3	0.63	0.11	0.44-0.89	0.63	0.12	0.43-0.91
	Adoptive - 4	1.45	0.48	0.75-2.78	1.47	0.50	0.75-2.88

Table S4. Results: Cause-specific Mortality for Swedish Men, Born 1915 to 1960. Model 1 adjusts for birth cohort. Model 2 adjusts for birth cohort, educational attainment, adult socioeconomic status, and origin of adopted children.

		Model 1			Model 2		
Cause-of-death	Parity	RR	SE	Cause-of-death	Parity	RR	SE
Neoplasms	Childless	1.00			1.00		
	Biological - 1	0.98	0.01	0.97-0.99	1.21	0.01	1.19-1.22
	Biological - 2	0.89	0.01	0.88-0.90	1.14	0.01	1.12-1.15
	Biological - 3	0.90	0.01	0.88-0.91	1.14	0.01	1.13-1.16
	Biological - 4	0.94	0.01	0.93-0.96	1.17	0.01	1.15-1.19
	Biological - 5	1.01	0.01	0.99-1.04	1.23	0.02	1.19-1.26
	Biological - 6	1.00	0.02	0.96-1.05	1.19	0.03	1.14-1.24
	Biological - 7	1.03	0.04	0.96-1.10	1.19	0.04	1.11-1.27
	Biological - 8	1.01	0.05	0.91-1.12	1.12	0.06	1.01-1.24
	Adoptive - 1	0.85	0.02	0.81-0.88	0.84	0.04	0.77-0.92
	Adoptive - 2	0.72	0.03	0.67-0.77	0.79	0.04	0.72-0.88
	Adoptive - 3	0.50	0.08	0.37-0.69	0.55	0.09	0.40-0.75
	Adoptive - 4	0.95	0.26	0.55-1.63	1.07	0.30	0.62-1.86
Diseases of the circulatory system	Childless	1.00			1.00		
	Biological - 1	0.77	0.00	0.76-0.78	0.98	0.01	0.97-0.99
	Biological - 2	0.66	0.00	0.65-0.66	0.89	0.00	0.88-0.90
	Biological - 3	0.68	0.00	0.67-0.68	0.91	0.00	0.90-0.92
	Biological - 4	0.76	0.01	0.74-0.77	0.97	0.01	0.96-0.99
	Biological - 5	0.83	0.01	0.82-0.85	1.04	0.01	1.02-1.06
	Biological - 6	0.89	0.01	0.86-0.92	1.08	0.02	1.05-1.12
	Biological - 7	0.94	0.02	0.90-0.99	1.11	0.03	1.05-1.16
	Biological - 8	0.92	0.04	0.86-0.99	1.03	0.04	0.96-1.11
	Adoptive - 1	0.67	0.01	0.65-0.70	0.68	0.03	0.62-0.74
	Adoptive - 2	0.48	0.02	0.45-0.51	0.54	0.03	0.49-0.59
	Adoptive - 3	0.45	0.06	0.35-0.58	0.48	0.07	0.37-0.63
	Adoptive - 4	0.67	0.17	0.41-1.12	0.69	0.18	0.41-1.15
External causes	Childless	1.00			1.00		
	Biological - 1	0.66	0.01	0.64-0.68	0.90	0.01	0.88-0.93
	Biological - 2	0.52	0.01	0.50-0.53	0.77	0.01	0.75-0.79
	Biological - 3	0.55	0.01	0.53-0.56	0.80	0.01	0.78-0.82
	Biological - 4	0.65	0.01	0.62-0.67	0.90	0.02	0.87-0.94
	Biological - 5	0.72	0.02	0.68-0.77	0.96	0.03	0.90-1.02
	Biological - 6	0.70	0.04	0.64-0.78	0.90	0.05	0.82-1.00
	Biological - 7	0.77	0.06	0.66-0.89	0.93	0.07	0.80-1.09
	Biological - 8	0.77	0.09	0.61-0.97	0.87	0.11	0.69-1.11
	Adoptive - 1	0.48	0.03	0.43-0.53	0.47	0.05	0.37-0.58
	Adoptive - 2	0.31	0.03	0.26-0.38	0.40	0.05	0.31-0.51
	Adoptive - 3	0.22	0.09	0.10-0.49	0.30	0.12	0.13-0.67
	Adoptive - 4	0.65	0.38	0.21-2.03	0.94	0.55	0.30-2.95
All other causes	Childless	1.00			1.00		
	Biological - 1	0.69	0.01	0.68-0.70	0.88	0.01	0.87-0.89
	Biological - 2	0.55	0.00	0.54-0.56	0.74	0.01	0.73-0.75
	Biological - 3	0.57	0.00	0.56-0.58	0.76	0.01	0.75-0.78
	Biological - 4	0.63	0.01	0.61-0.64	0.81	0.01	0.79-0.83
	Biological - 5	0.69	0.01	0.67-0.71	0.86	0.01	0.84-0.89
	Biological - 6	0.77	0.02	0.74-0.81	0.94	0.02	0.90-0.99
	Biological - 7	0.80	0.03	0.74-0.86	0.94	0.04	0.88-1.02
	Biological - 8	0.80	0.05	0.71-0.89	0.91	0.05	0.81-1.02
	Adoptive - 1	0.55	0.01	0.52-0.57	0.52	0.03	0.46-0.59
	Adoptive - 2	0.40	0.02	0.37-0.44	0.45	0.03	0.39-0.51
	Adoptive - 3	0.51	0.08	0.37-0.70	0.58	0.10	0.42-0.81
	Adoptive - 4	0.31	0.15	0.12-0.83	0.36	0.18	0.13-0.97

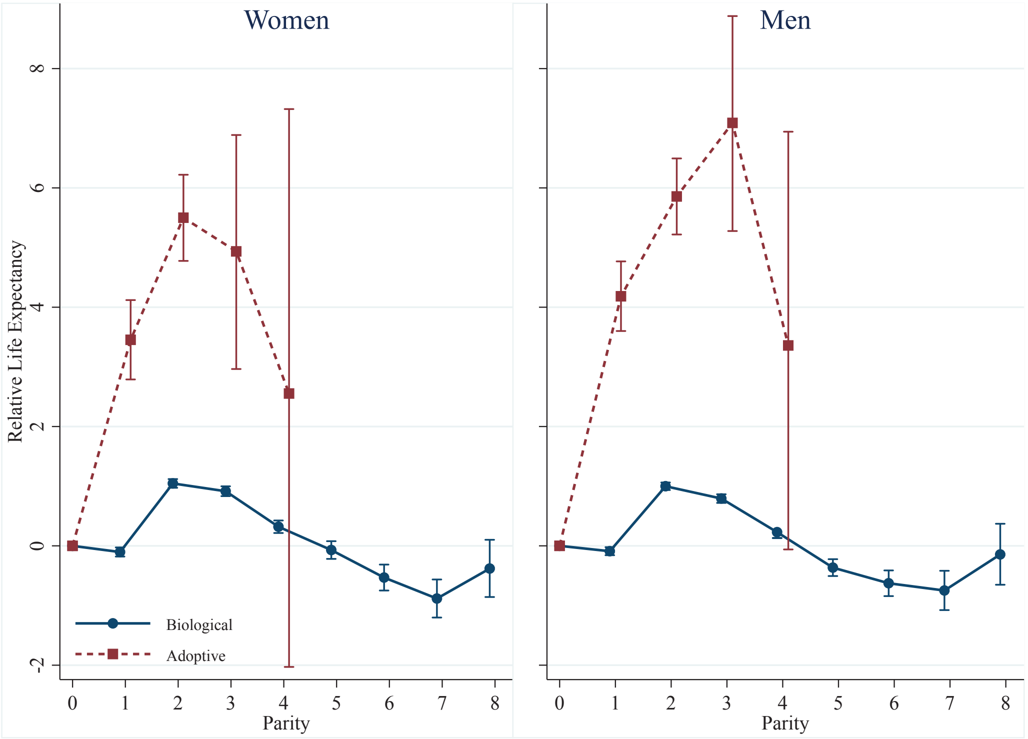
Women
Men
Relative Life Expectancy
Parity
Biological
Adoptive
-2
0
2
4
6
8
0
1
2
3
4
5
6
7
8
